# First experience with zero-fluoroscopic ablation for supraventricular tachycardias using a novel impedance and magnetic-field-based mapping system

**DOI:** 10.1007/s00392-018-1220-8

**Published:** 2018-02-23

**Authors:** Katie A. Walsh, Joseph Galvin, John Keaney, Edward Keelan, Gabor Szeplaki

**Affiliations:** 1 Heart and Vascular Centre, Mater Private Hospital, 72 Eccles Street, Dublin 7, Ireland; 2 Heart and Vascular Centre, Semmelweis University, Budapest, Hungary

**Keywords:** Supraventricular tachycardia, Ablation, Zero-fluoroscopy, Radiation, ENSITE precision, Typical atrial flutter

## Abstract

**Aims:**

Zero- and near-zero-fluoroscopic ablation techniques reduce the harmful effects of ionizing radiation during invasive electrophysiology procedures. We aimed to test the feasibility and safety of a zero-fluoroscopic strategy using a novel integrated magnetic and impedance-based electroanatomical mapping system for radiofrequency ablation (RFA) of supraventricular tachycardias (SVTs).

**Methods:**

We retrospectively studied 92 consecutive patients undergoing electrophysiology studies with/without RFA for supraventricular tachycardia (SVT) performed by a single operator at a single center. The first 42 (Group 1) underwent a conventional fluoroscopic-guided approach and the second 50 (Group 2) underwent a zero-fluoroscopic approach using the Ensite Precision^™^ 3-D magnetic and impedance-based mapping system (Abbott Inc).

**Results:**

Group 1 comprised 14 AV-nodal re-entrant tachycardia (AVNRT), 12 typical atrial flutter, 4 accessory pathway (AP), 2 atrial tachycardia (AT), and 9 diagnostic EP studies (EPS). Group 2 comprised 16 AVNRT, 17 atrial flutter, 6 AP, 3 AT, 2 AV-nodal ablations, and 7 EPS. A complete zero-fluoroscopic approach was achieved in 94% of Group 2 patients. All procedures were acutely successful, and no complications occurred. There was a significant reduction in fluoroscopy dose, dose area product, and time (*p* < 0.0001, for all), with no difference in procedure times. Ablation time for typical atrial flutter was shorter in Group 2 (*p* = 0.006).

**Conclusions:**

A zero-fluoroscopic strategy for diagnosis and treatment of SVTs using this novel 3D-electroanatomical mapping system is feasible in majority of patients, is safe, reduces ionizing radiation exposure, and does not compromise procedural times, success rates, or complication rates.

## Introduction

The lifelong risk of certain cancers is increased by the stochastic and non-stochastic effects of ionizing radiation. Fluoroscopy during radiofrequency ablation (RFA) of supraventricular tachycardia (SVT) exposes patients and operators to ionizing radiation. The demand for reducing radiation exposure by optimizing fluoroscopy or by the use of advanced technologies during these procedures is particularly important [1–4]. Previous reports have shown that the aim of zero-fluoroscopy during conventional ablation procedures can be reached [5]. In the present study, we aim to report the first experience which compares a zero-fluoroscopic strategy using a novel integrated impedance and magnetic-field-based electroanatomical mapping system with conventional fluoroscopic-guided strategies for RFA of SVT.

## Methods

### Study design

We retrospectively studied 92 consecutive patients who underwent electrophysiology studies (EPS) with or without RFA for SVT performed by a single operator at the Mater Private Hospital, Dublin, Ireland. The cases were a heterogenous mix of typical atrial flutter, AV-nodal re-entrant tachycardia (AVNRT), accessory pathway (AP), atrial tachycardia (AT), AV-nodal ablation, and diagnostic electrophysiological studies (EPS) for suspected SVT. The first 42 (group 1) underwent a conventional fluoroscopic-guided approach between 1 December 2015 and 30 June 2016. The second 50 (Group 2) underwent a zero-fluoroscopic approach using the novel Ensite Precision^™^3-D impedance-based mapping system which integrates magnetic data (Abbott Inc, USA), between 1 July 2016 and 30 April 2017.

### Procedure protocol

Procedures were performed under conscious sedation following the routine standards. Briefly, in case of a documented typical atrial flutter, a duo-decapolar catheter (Via Cath 20, Biotronik, Germany) was placed in the right atrium and a steerable decapolar catheter (Dynamic Deca, Boston Scientific, San Jose, CA, USA) was placed into the proximal coronary sinus (CS) in both groups. In case, where SVT was suspected, and a routine EPS was conducted to determine the arrhythmia mechanism standard quadripolar diagnostic catheters (Viking, Boston Scientific) were positioned to the high right atrium, His-bundle region and right ventricular apex, while a steerable decapolar catheter was positioned to the proximal CS.

**Fig. 1:**
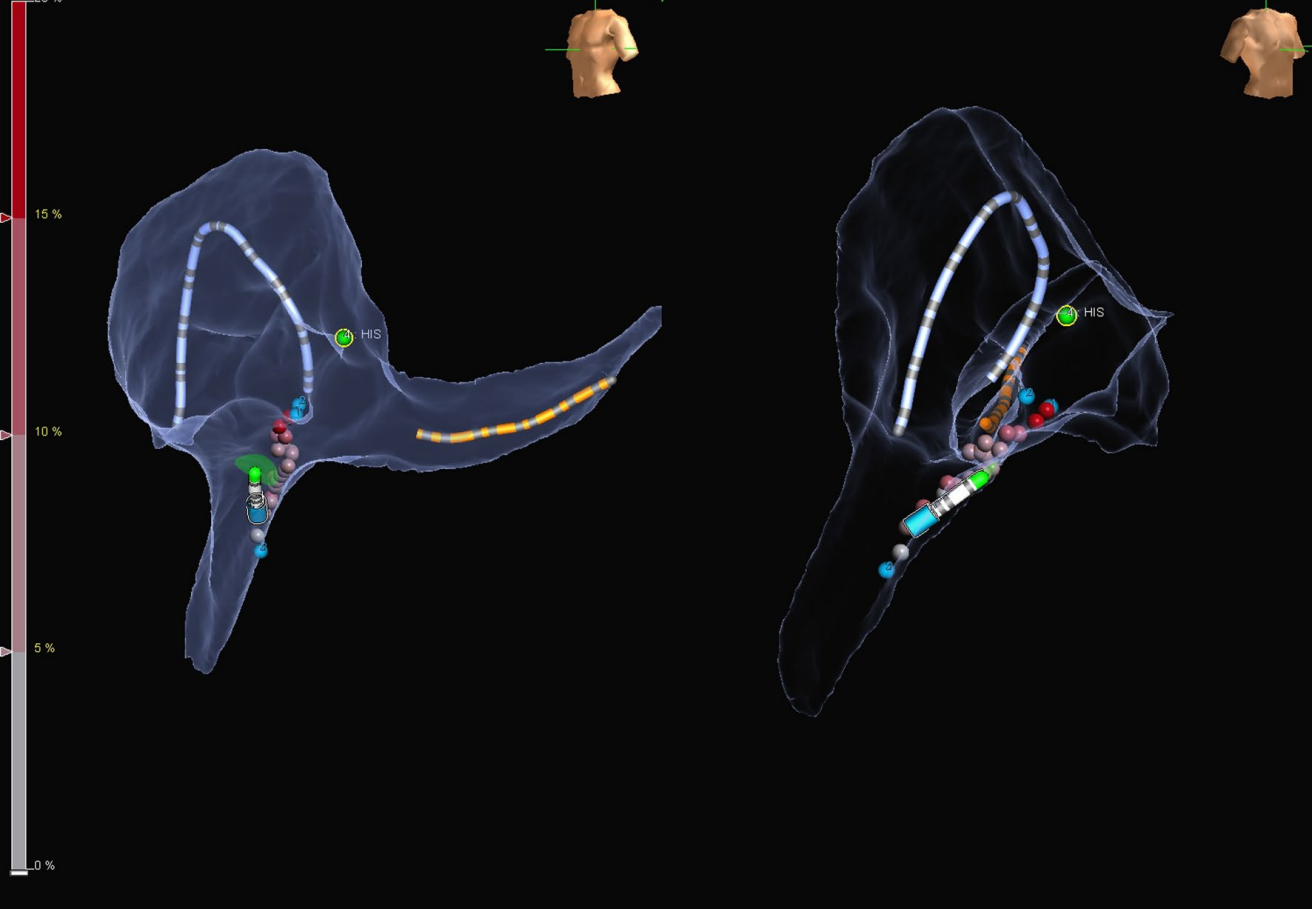
Representative electroanatomical map during ablation for typical atrial flutter in LAO (left panel) and RAO (right panel) projection. Catheters are placed by the aid of the mapping system using one bipole on the coronary sinus catheter as a reference. The geometry is created by the duo-decapolar diagnostic catheter and finalized by the ablation catheter, which enables to collect magnetic points. To avoid damage, the His region is tagged with green dots. The distal and proximal part of the cavo-tricuspid isthmus is marked by blue dots, while different shades of red represent the ablation tags

**Fig. 2:**
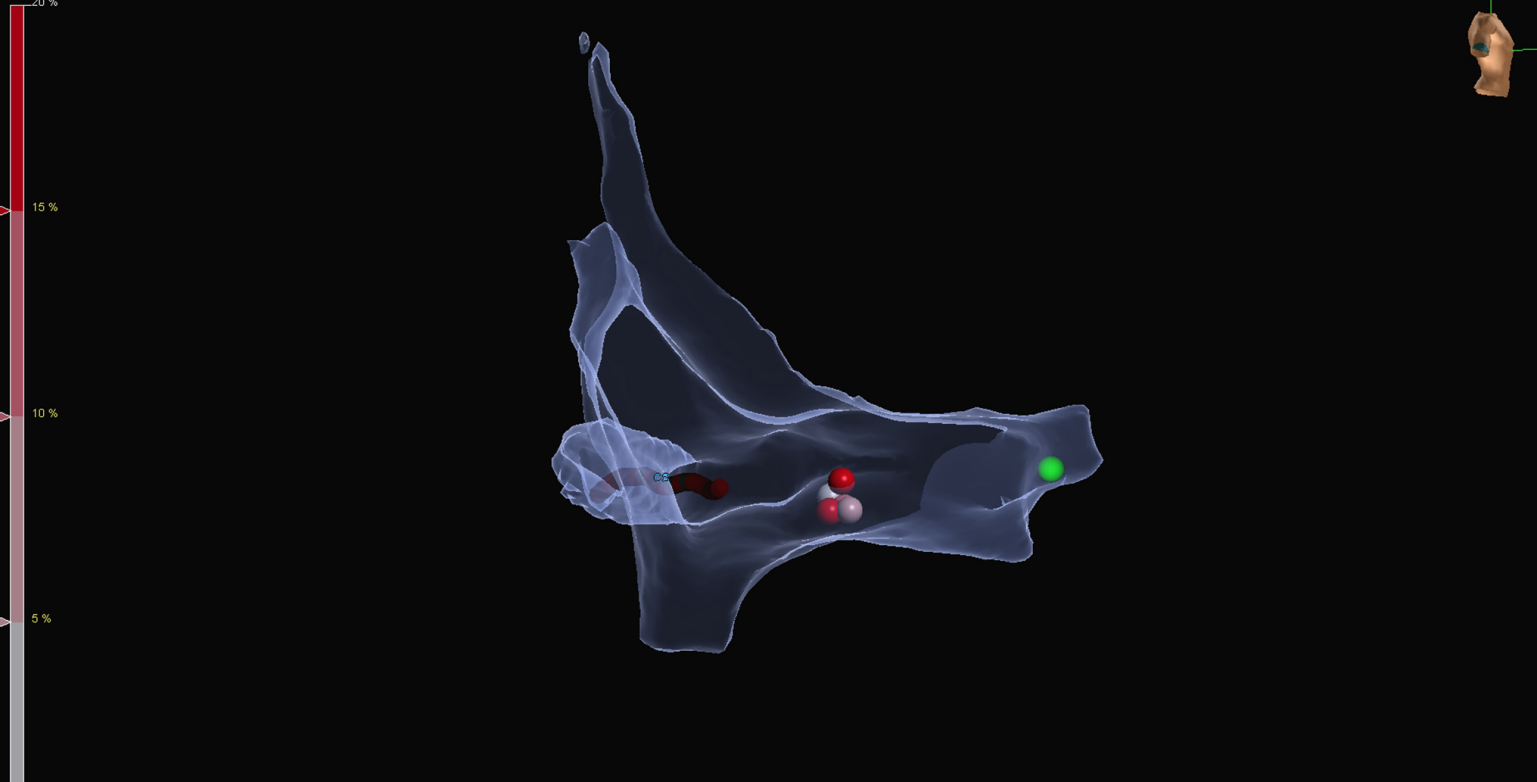
Representative electroanatomical map during slow pathway ablation for AV-nodal re-entrant tachycardia (right lateral view). Catheters are placed by the aid of the mapping system using one bipole on the coronary sinus catheter as a reference. The relevant part of the right atrium is mapped with the sensor enabled ablation catheter. The green tag represents the His region, while red tags represent ablation points at the slow pathway region

Catheter placement was done under fluoroscopy guidance in Group 1, whereas using zero-fluoroscopy approach in Group 2. In Group 1, ablation for typical atrial flutter was performed using the standard FlexAbility^™^ (Abbott Inc) irrigated catheter in a temperature limited power controlled mode set to 35 W and 43 °C, while a Celsius (Biosense Webster) non-irrigated catheter was used for non-flutter case set at 40W and 65 °C. In Group 2, ablation was performed using the FlexAbility^TM^-sensor-enabled^™^ (Abbott Inc) irrigated catheter at 35 W and 43 °C. The Ensite Precision (Abbott Inc) magnetic and impedance-based 3D electroanatomical mapping system (EAM) was used in Group 2 to create an electroanatomic cardiac shell. The position of the His bundle was recorded and tagged on the shell. Representative maps of the two most common arrhythmias illustrating the method are shown in Figs. 1 and 2 (typical atrial flutter and AVNRT, respectively). The ileo-femoral venous system and inferior vena cava were visualised by the EAM system to facilitate catheter placement from the right femoral vein. Laboratory staff did not wear lead protection for the zero-fluoroscopy cases, and were only used when conversion to radiation was required.

Patient demographics, the details of the arrhythmias, and ablation data were retrospectively collected. The study complied with the Declaration of Helsinki and the study protocol was approved by the hospital research ethics committee (ref: 1/378/1908 TMR, Mater Misericordiae University Hospital, Dublin, Ireland).

### Statistical analyses

Statistical analyses were performed using IBM SPSS statistical software package, version 24 (Apache Software Foundation, USA). As most of the parameters had non-Gaussian distributions, nonparametric tests were used throughout the analysis (tested by the Kolmogorov Smirnov test). All tests were performed two-tailed with a significance level set to *p* < 0.05. Continuous variables are reported as medians and interquartile ranges; categorical variables are reported as absolute numbers (%). The Mann–Whitney *U* test was used to test for differences between the groups for continuous variables, while the Fisher`s Exact test for categorical variables.

## Results

### Success of the zero-fluoroscopic approach

There were no baseline differences between the two patient groups regarding age, co-morbidities, and types of procedures performed (Table 1). A complete zero-fluoroscopic approach was successfully performed in 47/50 (94%) of Group 2 cases. Conversion to minimal fluoroscopic approach was required in three cases, only for performing trans-septal puncture in patients with arrhythmias requiring left atrial access (2 concealed left lateral accessory pathways and 1 left atrial tachycardia). One trans-septal catheterization was performed with zero-fluoroscopy, as the catheter was passed through a residual trans-septal defect from a prior procedure in a case of recurrence of a concealed left lateral accessory pathway and 1 intracardiac echocardiography (ICE) guided trans-septal puncture was done successfully with the zero-fluoroscopy approach. All right-sided procedures were successfully done using the zero-fluoroscopy approach, without the need for conversion to fluoroscopy guidance.

**Table 1:** Descriptive statistics of the study population

	Group 1 Conventional *N* = 42	Group 2 Zero-fluoroscopy *N* = 50	*p* value
Male/female	25/17 (59.5%/40.5%)	30/20 (60.0%/40.0%)	1.000
Age	56 (36–69)	66 (49–74)	0.054
Co-morbidities			
Ischemic heart disease	2 (4.8%)	5 (10.0%)	0.448
Hypertension	7 (16.7%)	7 (14.0%)	0.777
LV dysfunction	3 (7.1%)	4 (8.0%)	1.000
Diabetes mellitus	2 (4.8%)	3 (6.0%)	1.000
EPS findings			
Typical atrial flutter	12 (28.6%)	17 (34.0%)	0.655
AVNRT	14 (33.3%)	16 (32.0%)	1.000
Accessory pathway	4 (9.5%)	6 (12.0%)	0.750
Atrial tachycardia	3 (7.1%)	2 (4.0%)	0.654
AV-nodal ablation	0 (0%)	2 (4.0%)	0.495
Diagnostic EPS	9 (21.4%)	7 (14.0%)	1.000

Values presented as absolute numbers (%) or medians (interquartile ranges)

*LV* left ventricular, *EPS* electrophysiology study, *AVNRT* AV-nodal re-entrant tachycardia

### Procedural success, complications, and arrhythmia recurrence

All procedures were acutely successful and none of the patients in either group had acute or late complications. At follow-up with a median 4.9 (1.7–9.3) months, one single patient in Group 2 had a recurrence of the index arrhythmia and required a repeat procedure. This patient had an atrial tachycardia, which was ablated from the non-coronary cusp. The repeat ablation procedure for that patient was done with high-density mapping and with the use of fluoroscopy. After the second ablation procedure (similarly to the index procedure), the patient was asymptomatic for a few weeks, but unfortunately experienced a further recurrence. With antiarrhythmic drug therapy, the patient is now free of sustained tachycardia episodes. There was no arrhythmia recurrence in Group 1.

### Procedural parameters

Radiation dose, dose area product (DAP), and fluoroscopy time, as well as procedure and ablation times were compared between the two study groups (Table 2). In Group 2 (zero-fluoroscopy) radiation dose, DAP and fluoroscopy time were significantly decreased compared with Group 1 (*p* < 0.0001, for all), with no difference in procedure and ablation times. As the majority (64%) of the patients had either typical atrial flutter or AVNRT, we did a subgroup analysis for those arrhythmias and compared the two groups. Figures 1 and 2 show some illustrative images obtained during ablation of those arrhythmias. Our results have shown (see Tables 3, 4) that in addition to the decreased radiation dose, DAP and fluoroscopy time in both groups, there was a significant reduction in ablation time in patients with typical atrial flutter (*p* = 0.006).

**Table 2:** Procedural parameters in the study population

	Group 1 Conventional N = 42	Group 2 Zero-fluoroscopy N = 50	*p* value
Fluoroscopy dose (mGy)	13.5 (4.8–33.5)	0 (0–0)	< 0.001
Dose area product (µGy m^2^)	117.4 (44.1–338.6)	0 (0–0)	< 0.001
Fluoroscopy time (min)	6.25 (3-12.7)	0 (0–0)	< 0.001
Procedure time (min)	49 (44–70)	57 (44–72)	0.180
Ablation time (s)	134 (58–545)	133.5 (69–274)	0.690

Values presented as medians (interquartile ranges)

**Table 3:** Procedural parameters in patients with typical atrial flutter

	Group 1 Conventional *N* = 12	Group 2 Zero-fluoroscopy *N* = 17	*p* value
Fluoroscopy dose (mGy)	35.5 (15.0–53.6)	0 (0–0)	< 0.001
Dose area product (µGy m^2^)	389.5 (131–625.5)	0 (0–0)	< 0.001
Fluoroscopy time (min)	9.7 (6.2–21.1)	0 (0–0)	< 0.001
Procedure time (min)	49 (46–88)	49.5 (44.5–59.5)	0.732
Ablation time (s)	753.3 (438–1459.5)	279.5 (148.5–500.5)	0.006

Values presented as medians (interquartile ranges)

**Table 4:** Procedural parameters in patients with AV-nodal re-entrant tachycardia

	Group 1 Conventional N = 14	Group 2 Zero-fluoro N = 16	*p* value
Fluoroscopy dose (mGy)	10.2 (4.5–25.3)	0 (0–0)	< 0.001
Dose area product (µGy m^2^)	85.2 (44–195)	0 (0–0)	< 0.001
Fluoroscopy time (min)	4.95 (3.2–12.3)	0 (0–0)	< 0.001
Procedure time (min)	49 (46–56)	57.5 (47–66)	0.294
Ablation time (s)	99.5 (61–151)	100 (63–151)	0.950

Values presented as medians (interquartile ranges)

## Discussion

### Main findings

In this series, zero-fluoroscopic approach was achieved in 94% of cases with the use of the novel integrated impedance and magnetic-field-based electroanatomical mapping system. We reported a significant reduction in fluoroscopy doses almost to zero in this population, without compromising procedure times, success rates, or complications.

This is the first series with the novel EnSite Precision^™^ EAM system, which illustrated the feasibility, safety, and benefits of use of the to achieve a fully zero-fluoroscopic approach in the vast majority of SVT cases. In our experience, the approach was feasible in all cases, where right atrial access was required. In three patients, fluoroscopy was required only to aid trans-septal puncture. However, it must be noted that we reported on the first consecutive 50 cases we have performed using this technique, which involved the learning curve, that might have had a bias on the trans-septal punctures. With the increasing operator experience following the learning curve and the use of ICE, we managed to overcome that limitation as well. Alternatively, trans-oesophageal echocardiography might be helpful in those cases to facilitate the trans-septal puncture. Thus, zero-fluoroscopy approach can be feasible for SVTs that require left atrial access as well.

In our cohort, significantly less ablation was required to achieve cavo-tricuspid isthmus block in typical atrial flutter, using the novel approach. Minimizing ablation time for these cases might have the potential to reduce ablation-related complications.

### Risks of radiation exposure

The risk of radiation exposure in cardiology is well recognized for patients and as an occupational hazard for laboratory staff [6]. Medical exposure to radiation has increased in line with advancements in diagnostic imaging and is now the most significant manmade source of radiation [7]. In 2006, medical exposure constituted nearly half of the total radiation exposure of the US population from all sources [8]. Cardiology procedures account for about 40% of the entire cumulative effective radiation dose to the US population from all medical sources, excluding radiotherapy and any attempt to reduce exposure is essential [1, 9–11].

Radiation increases the lifetime risk of certain carcinomas, via stochastic and non-stochastic (deterministic) effects. The latent period between radiation exposure and cancer presentation confers that younger patients are more susceptible to this risk (as in elderly patients, this latent period is more likely to exceed the patient’s life expectancy). This is important in electrophysiology as many patients undergoing SVT ablation are relatively young with few co-morbidities, and SVT ablation is common also in the paediatric population [12].

It is not just patients who are at risk, but operators too; a growing body of evidence exists implicating radiation exposure in vascular disease [13], cognitive impairment [14], and tumours of the brain and neck [15] in physicians who perform fluoroscopic-guided interventional procedures. Furthermore, wearing lead protection has been associated with fatigue and orthopadic complaints for laboratory staff [16], although lead aprons block just about one-third of scattered radiation [17]. Given these well-recognized hazards, it is vitally important that zero- or near-zero-fluoroscopic approaches in EP are explored and refined, to minimise risks. These are especially important in high-risk populations, including children, pregnant women, and women with child-bearing potential [18, 19].

### Previous studies

The benefits of using 3D electroanatomic mapping (EAM) systems to minimise radiation in the electrophysiology lab have been documented in several recent reports of minimal and zero-fluoroscopy approaches during SVT ablation [5]. Current systems in use include the EnSite NavX, the MediGuide (Abbott Inc, both), the CARTO 3 (Biosense Webster, Diamond Bar, CA, USA), and the Rhythmia (Boston Scientific, San Jose, CA, USA). The CARTO-UNIVU^™^ Module (Biosense Webster, Diamond Bar, CA, USA) merges real-time EAMs with pre-acquired fluoroscopy images to help reduce overall radiation use. These mapping systems mainly rely on combinations of magnetic location technology with impedance-based data for catheter localization.

Current evidence supports the assumption that 3D-EAM systems reduce fluoroscopy exposure without affecting procedure safety and outcomes [5]. Success rates using these approaches are variable, but generally are high (70–95%) for the diagnosis and ablation of SVTs [5] (where success is defined as complete zero-fluoroscopy during the procedure). Success rates are certainly depending on the type of the procedure, operator experience and patient selection. The success rates for complete zero-fluoroscopy were reported around 80% for AVNRT in an early publication [20] and 90% for typical atrial flutter in a recent one [21]; it was almost 95% in a population with right-sided accessory pathways [22]. Our result of an overall 94% success in complete zero-fluoroscopy (and 100% for right-sided procedures) is well within the desired range of success and is in line with previous observations.

Although most of the data on zero-fluoroscopy are derived from single-center experiences, an Italian multi-center trial (NO-PARTY) randomized 262 patients with SVTs to the EnSite^TM^NavX^™^ navigation system with minimal fluoroscopy or a conventional approach. Zero-fluoroscopy was achieved in 72% of patients in the minimal fluoroscopy group, with significant overall reduction of the radiation dose and associated late risks. Moreover, a cost-effectiveness analysis was also performed with a recommendation on acceptable extra-costs in the same series [23]. Moreover, a prospective, randomized study using the MediGuide system has also confirmed significant radiation exposure reduction, without affecting success and complication rates (although this latter method requires fluoroscopy at the beginning of the study, and, therefore, is not entirely zero-fluoroscopic) [24].

Literature data on late effects of ionizing radiation and cancer incidence during electrophysiology procedures are scarce. Authors of the randomized NO-PARTY trial assessed lifetime attributable risks of cancer incidence and mortality from equivalent organ doses calculated with Monte Carlo code, according to the Biological Effects of Ionizing Radiation empirical risk models [23]. According to their results, the lifetime attributable cancer incidence ranged between 7.3 (95% confidence interval: 3.4–12.8)–11.0 (6.0–18.6) for males and 8.2 (5.0–12.8)–15.4 (9.9–25.3) for females for minimal fluoroscopy approach and 195 (111–315)–321 (198–512) and 241 (165–350)–486 (333–773) for females for the conventional approach, per 100 000 individuals (depending on age). For lifetime attributable cancer mortality risk, their results showed 3.7 (1.5–6.9)–4.8 (2.5–8.2) for males and 4.1 (2.3–6.7)–6.1 (3.9–9.2) for females for minimal fluoroscopic approach and 94 (49–158)–136 (82–215) for males and 115 (76–171)–186 (131–265) for the conventional approach, per 100 000 individuals (depending on age). Overall, minimal fluoroscopic approach resulted in 96% reduction of lifetime attributable cancer risks, although complete zero-fluoroscopy was reached in 72% of the cases (compared with 94% reported here). Similarly, they have also calculated years of life lost and years of life affected due to predicted long term. Radiation during conventional procedures would account for a total of 32 years of life lost in a sample population of 1000 woman, aged 15 years, according to their results [23].

In addition to reduction in fluoroscopy dose, our study also confirmed less ablation time required in the typical atrial flutter subgroup. The positive effect observed on minimizing radiation confirms the previous observations [21, 24–27]; however, the one on reduced ablation time extends those further. The difference of the ablation times did not relate to different catheter technologies, as in the typical atrial flutter subgroup similar catheters were used (FlexAbility^™^, Abott Inc). The only difference in the catheter was the presence or absence of the magnetic sensor, which presumably did not affect ablation parameters. However, the two different strategies we used clearly differed and not only in the use or absence of radiation, but also in the availability of EAM during the procedures. It is known that the use of 3D EAM systems might be helpful in the ablation of typical atrial flutter [26]. The reduced ablation time might more likely be related to the EAM, rather than the lack of fluoroscopy with the novel approach. However, as no impact on ablation time was reported with the earlier version of the EAM system [28], our observation might relate to the improved navigation capacity and stability of the novel 3D EAM system. Certainly, this observation and assumption needs further clinical validation.

The EnSite Precision^™^ system employs a combination of both magnetic and impedance field data to allow EAM and real-time localization of multiple catheters, with the capability to map any cardiac chamber with any catheter. The use of magnetic points with Sensor Enabled^™^ catheters serves to correct impedance distortion and helps to maintain the anatomic accuracy of the geometry in the map. Using a combination of both impedance and electromagnetic technologies, the system achieves a coordinate system accuracy of 2 mm. The simultaneous collection of anatomic and electrical points from multiple electrodes leads to a significantly faster point collection vs. manual mapping [29] and superiority in high point density through the creation of 3-D models [30]. According to our first experience, the system is feasible and safe to use for a zero-fluoroscopic approach of SVTs, which results in a significant reduction of radiation exposure to the patients and staff both.

## Limitations

Our series is of a single operator in a single center study, which may limit its applicability in other centers. The study is retrospective, non-randomized with limited number of patients included. Different types of catheters were used in the two groups, which were related to the retrospective design. For flutter ablation, the use of different technologies might have biased the observed difference in ablation times. There may have been a learning curve effect influencing the procedure times and the ability to perform fluoroless trans-septal punctures for the first cases in Group 2. The numbers of patients in the subgroup analyses are low. These procedures generally carry low complication rates and the study might not be sufficient to show differences between the two groups, especially in cardiac perforation. All results should be considered as hypothesis generating and further large scale, multi-center studies with longer follow-up duration periods are required to validate the results.

## Conclusions

This study adds to the growing experience with minimal and zero-fluoroscopic approaches for SVT ablation. Our series identifies for the first time in a consecutive series the safe and feasible role of the EnSite Precision^™^ impedance and magnetic-based mapping system in achieving the goal of zero-fluoroscopic ablation. It dramatically reduces radiation dose, without compromising procedure times. Furthermore, this approach avoids the disadvantages of heavy lead protection and might prevent orthopedic hazards as well. These are encouraging data for the future and another step toward achieving complete zero-fluoroscopy ablation.
